# Comparative study of bioclimatic conditions in southwestern Greenland between the late 18th century and the present

**DOI:** 10.1007/s00484-026-03132-5

**Published:** 2026-03-04

**Authors:** Andrzej Araźny, Konrad Chmist, Rajmund Przybylak, Przemysław Wyszyński, Garima Singh

**Affiliations:** 1 Faculty of Earth Sciences and Spatial Management, Nicolaus Copernicus University in Toruń, Toruń, Poland; 2 Centre for Climate Change Research, Nicolaus Copernicus University, Toruń, Poland

**Keywords:** Greenland, Historical bioclimatology, Moravian brethren, Wind chill temperature, Insulation predicted

## Abstract

The paper compares the scope and nature of changes in meteorological and biometeorological conditions in Nuuk (SW Greenland) in the second half of the 18th century for “expedition years” (Sep–Aug of: 1767–68, 1789–90, 1790–91 and 1791–92) against the contemporary period (1991–2020). The analysis used air temperature, wind speed and biometeorological indices: wind chill temperature (WCT) and insulation predicted (Iclp). The analysis was performed for individual days and months, and the comparison covers mainly the period January–June, for which complete data are available. The study showed that, in the years 1789–92, the prevailing conditions according to WCT were very similar to the contemporary period. However, according to WCT, 1768 was 4.5 °C milder for humans compared to current prevailing conditions for the period January–June. These relationships are also confirmed by the analysis of the Iclp index. Weather conditions were decidedly less burdensome for humans in 1768; for example, a person on the move needed about 0.3 clo less clothing than today. This paper is the first historical bioclimatology study of weather conditions for humans in 18th-century Greenland.

**Supplementary Information:**

The online version contains supplementary material available at 10.1007/s00484-026-03132-5.

## Introduction

People feel the effects of the rapidly changing Arctic through a combination of environmental factors. These include changes in physical conditions, how biological resources respond, impacts on infrastructure, decisions that affect our ability to adapt, and both environmental and international influences on the economy and overall well-being (SEARCH et al. 2022). The Arctic plays a very important role in shaping the climate and living conditions of lower latitudes (Serreze and Barry 2014; Przybylak 2016). Air temperature in the Arctic rise twice as fast as in any other region in the world and is transitioning to a new Arctic climate state (Landrum and Holland 2020; Przybylak and Wyszyński 2020; IPCC 2021; Wang 2023). The Arctic has unfavorable bioclimatic conditions, and every day a person can be exposed to the effects of low temperatures and strong wind (e.g., Gavhed 2003; Araźny 2008, 2019; Araźny et al. 2019; Kim et al. 2025).

Greenland occupies a crucial position within the Earth’s climate system. Its extensive landmass, covered by an ice sheet in the North Atlantic region, exerts a significant influence on global atmospheric circulation patterns (Abermann et al. 2017). The climate of Greenland’s south-western coast has been studied since the 18th century (Vinther et al. 2006; Borm et al. 2021). The first observations and measurements of weather conditions in Greenland were performed by Moravian missionaries beginning in 1733 (Hutton 1922; Lüdecke 2005). The reliability and unique nature of the meteorological observations conducted by the Moravian Church will significantly improve knowledge of the climate of Greenland and the Arctic during that period. The literature contains selected analyses of some series of 18th-century meteorological observations for the study area (e.g., Vinther et al. 2006; Demarée et al. 2020; Demarée and Ogilvie 2021; Przybylak et al. 2024). However, there is a noticeable lack of studies on human bioclimatology in Greenland in historical times. This study fills that gap. It allows, for the first time, a complete and reliable assessment of weather conditions from the point of view of human bioclimatology in 18th-century Greenland.

The only studies of bioclimatic conditions in the Arctic for historical times (from the 18th to the early 20th century) were made for: (1) stations operating in the Arctic during the First International Polar Year in 1882/83 (Araźny 2010); (2) the Franz Josef Land archipelago (Russian Arctic) in the late 19th and early 20th centuries (Araźny et al. 2019) and (3) recently by Chmist et al. (2025) for the north-eastern part of the Labrador Peninsula in the second half of the 18th century. More research works on the influence of weather and climate on humans were written in the 20th and 21 st centuries. They mainly concern the Norwegian Arctic area (e.g., Nordli et al. 2000; Araźny 2006, 2008; 2010, 2012, 2019; Araźny and Błażejczyk 2007; Araźny et al. 2009, 2010, 2019; Sikora et al. 2010; Maciejczyk et al. 2017) and higher-latitude areas of North America (e.g., Keiming and Bradley 2002; Mekis et al. 2015; Howarth and Laird 2017; Grigorieva et al. 2023; Kim et al. 2025).

In this article, we present a comprehensive analysis of bioclimatic conditions in south-western Greenland in the second half of the 18th century based on measurements made by Moravian missionaries. Biometeorological indices were calculated using early instrumental meteorological data that are the oldest existing weather series not only for Greenland but also for the entire Arctic. The main aim of the article is to analyze the bioclimatic conditions for humans in Greenland in Nuuk in the second half of the 18th century and to estimate the differences compared to the present conditions in this region.

## Research area, materials and methods

**Fig. 1:**
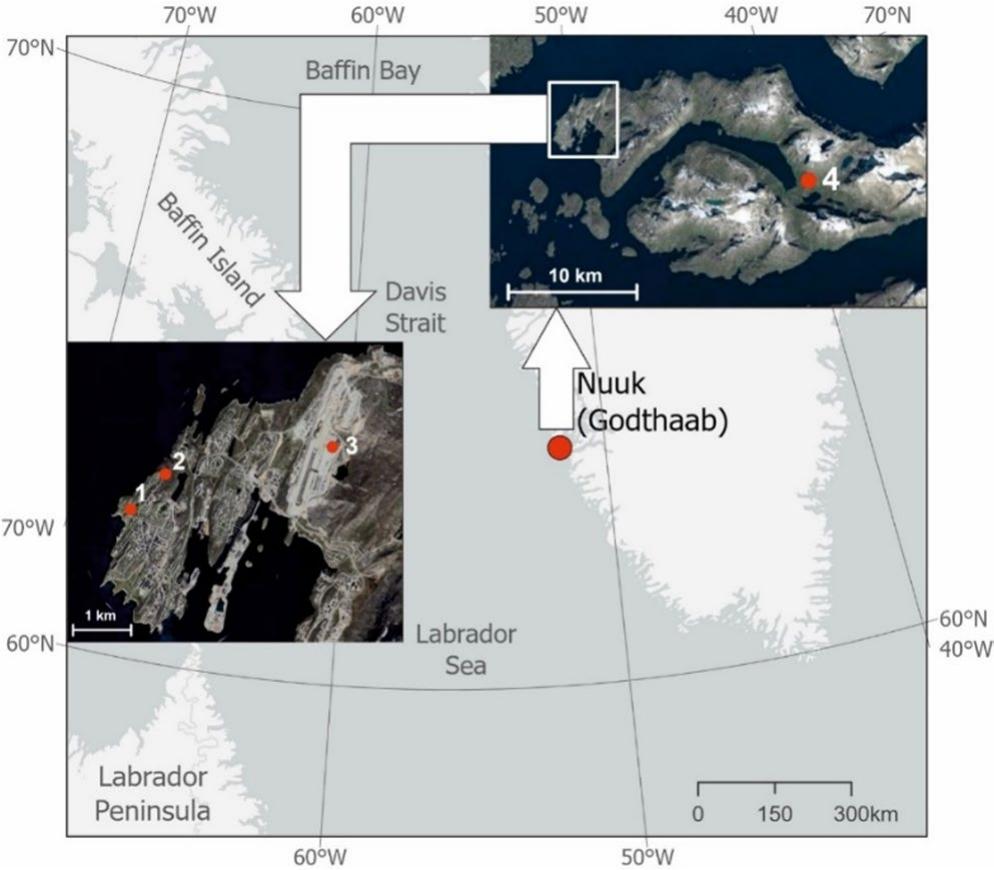
Locations of the study site and historical and contemporary measurement points Explanation: 1 – historical sites Neu-Herrnhut (1767–68) and Godthaab (1789–92); 2–4250 Nuuk (1991–2020); 3–4254 Mittarfik Nuuk (2001–20); 4 – Pissiksarbik. Map data for location of sites: © Google Earth; images © 2023 Maxar Technologies, © 2023 Airbus and © 2023 Asiaq

Nuuk (former names of the settlement: Godthaab, Godthåb or Godthab, and Neu-Herrnhut) is located in the south western part of the island, on the Davis Strait, about 250 km south of the Arctic Circle (Fig. 1). It is the oldest Danish settlement in Greenland, founded in 1721 on the island of Kangeq by the Danish–Norwegian missionary Hans Egede (Mills 2003). After a few years, Egede found the windy climate of Kangeq to be less than ideal and relocated its operation to Godthåb (Good Hope) in 1728, a few kilometers further up the Fjord (Nuttal 2005; Dzik 2018). The study area was originally inhabited by the Inuit in the 18th century. They were part of aspects of the life of the Moravian Brethren who arrived in Greenland in 1733 (Hutton 1922; Lüdecke 2005). In that same year, they established their own mission station, Neu-Herrnhut, in the immediate vicinity of Egede’s Godthåb colony (Nuttal 2005). Both places are located within the present-day settlement of Nuuk. The Inuit greatly helped the Moravian Brethren acclimatize to life in the harsh conditions of the barren lands and to learn about the landscape and local climatic conditions.

Today, Nuuk plays an important role as the administrative, cultural and economic centre of Greenland. Nuuk lies on the west coast of the island and is one of the world’s northernmost major cities and ports. According to the Köppen–Geiger climate classification, Nuuk has a maritime-influenced tundra climate (ET) (Kottek et al. 2006). The climate of Nuuk is characterized by long, cold, snowy winters and short, cool summers. The average annual air temperature at Nuuk from 1991 to 2020 was 1.0 °C. Over the course of the year, the average air temperature ranged from ˗8.3 °C in February to 7.0 °C in July (Cappelen and Drost Jensen 2021). The average annual precipitation at Nuuk was 874.0 mm. The lowest rainfall (53.0 mm) was recorded in April and the highest (106.0 mm) in September. The average annual wind speed at Nuuk, according to climatological standard normals from 1991 to 2020, was 6.0 m∙s^−1^ (Cappelen and Drost Jensen 2021).

**Fig. 2:**
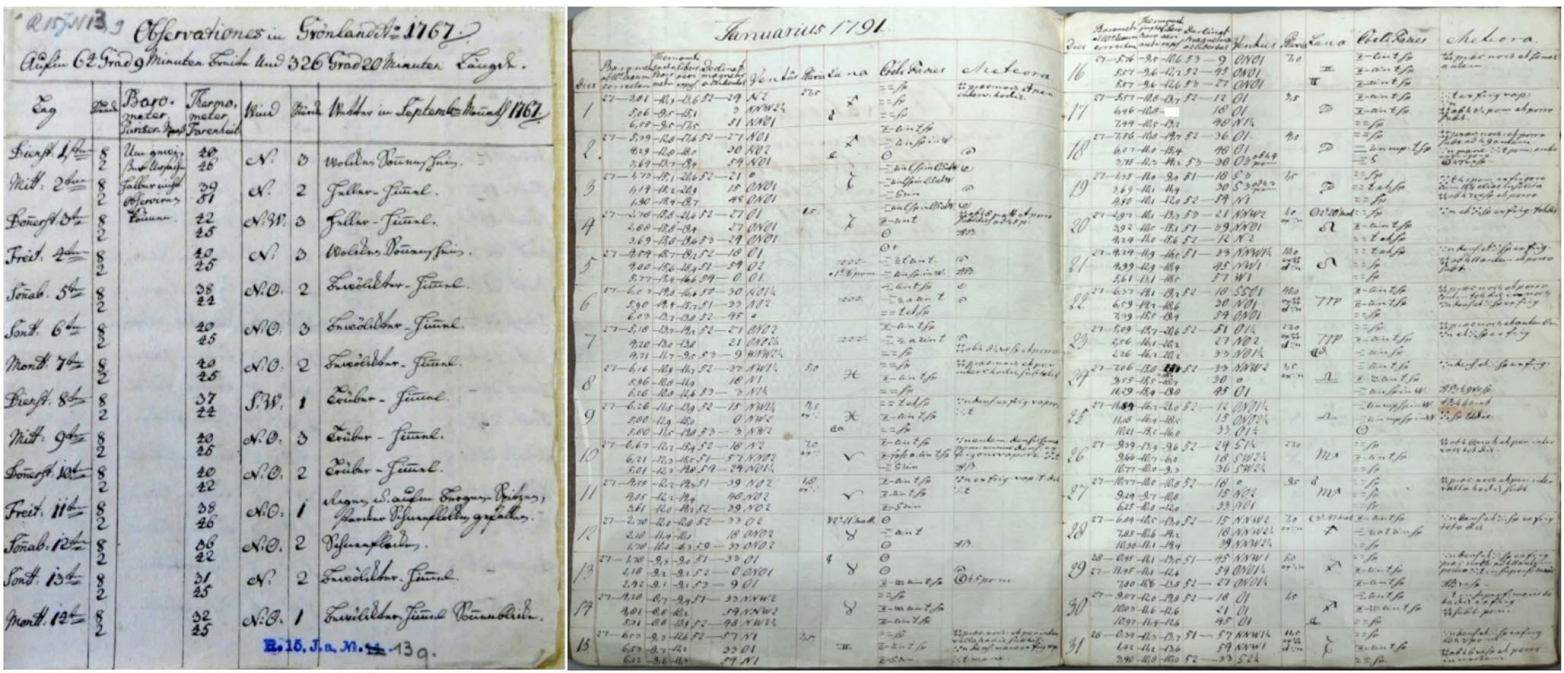
Examples of manuscripts presenting meteorological observations: (photo on the left) for Neu-Herrnhut (1 September 1767 to 22 July 1768), source – MH R.15 J.a.13.9 (Moravian Archives in Herrnhut; data presented in the manuscript: 1–14 September 1767), and (photo from the right) for Godthåb (1782–92), source – *Astronomiske og meteorologisk Iagttagelser*,* anstillede i Godthaab i Grønland 1782–92* (Det Kgl. Bibliotek in Copenhagen; data presented in the manuscript: January 1791)

The bioclimate in Nuuk region was estimated using two existing series of meteorological observations from stations located in this area: Neu-Herrnhut (1st Sep 1767–22nd Jul 1768) and Godthåb (January 1790 – June 1792). The location of measurement sites in the Nuuk region changed slightly (Fig. 1), e.g. the place of observations was changed in the beginning of June 1768 to Pissiksarbik (Crantz 1820). For more detail, see Przybylak et al. (2024). The first series is the oldest available long-term series of instrumental measurements for this region. Observations of air temperature, atmospheric pressure, and wind direction and force were made twice a day by Christopher Brasen (1738–74) at 08:00 and 14:00 LT. Data from this period were obtained from Moravian Archives in Herrnhut (catalogue number MH R.15 J.a.13.9, Fig. 2). The second series of measurements covers the period January 1784 – July 1792, but, for the first years, wind speed measurements are either lacking or of low quality (for details see Przybylak et al. 2024). Therefore, based on available data from this period, it is possible to calculate biometeorological indices only for the expeditions years (1789–90; 1790–91 and 1791–92). Data from this period were found in The Royal Library in Copenhagen in the manuscript *Astronomiske og meteorologisk Iagttagelser*,* anstillede i Godthaab i Grønland 1782–1792* (Fig. 2). Data and information on air temperature and wind force were transcribed from source materials and digitized for this study. This approach guarantees the lowest error rates when acquiring data from antique books and prints (Brönnimann et al. 2006).

Meteorological and biometeorological conditions were assessed for 14:00 Local Time (LT), which represents the most comfortable conditions for humans, i.e. early afternoon. The historical results were compared with contemporary ones from 1991 to 2020 for 15:00 LT, which is the closest available time of day to the historical data for 14:00 LT (Danish Meteorological Institute, https://www.dmi.dk). The original air temperature observations in Fahrenheit (°F) has been converted to Celsius (°C). Visual assessments of wind force during the historical period were made using scales of 1–6 and 0–4, for the periods 1767–68 and 1789–92, respectively. These values, when analyzing the descriptions presented for their characteristics (see Table 1S), refer to sea conditions, so they were first converted to the Beaufort scale (defined in 1806), and the corresponding wind speed values were then adopted according to WMO-No 306 (2019). The procedure for converting wind force from the scales used by the Moravian Brethren in the historical period to m·s^˗1^ is detailed by Chmist et al. (2025). The transformation was as follows: 1–0.45 m·s^˗1^, 2–3.4 m·s^˗1^, 3–8.0 m·s^˗1^, 4–13.9 m·s^˗1^, 5–20.75 m·s^˗1^, 6–30.5 m·s^˗1^. For the years 1789–92, the transformation was: 0–0.3 m·s^˗1^, 1–2.4 m·s^˗1^, 2–9.3 m·s^˗1^, 3–18.9 m·s^˗1^, 4–30.5 m·s^˗1^. The given values correspond to wind speed at a height of 10 m above sea level, which relates to the use of Beaufort scale. Therefore, they were then recalculated to a height of 1.2 m above sea level, because bioclimatological studies usually employ wind speed at the height of a person’s chest (e.g., in calculating Iclp). The obtained values (both historical and contemporary) were recalculated using the formula (Liopo and Cicenko 1971):

$$\:V_z=V_w\left(\frac{h_z}{h_w}\right)^{0.2}$$

where: Vz – wind speed at the height hz = 1.2–10 m, Vw – wind speed at the height of the anemometer hw – wind speed at the initial height (m∙s^˗1^).

Apparent conditions in Nuuk are presented using two biometeorological indices: wind chill temperature (WCT) and insulation predicted (Iclp). They were calculated using the BioKlima 2.6 software package (2024) (https://www.igipz.pan.pl/BioKlima.html).

The wind chill temperature (WCT) index is used to assess the perceived air temperature and the risk of frostbite, mainly of exposed body parts, as well as the impact of the prevailing conditions on human health (www.canada.ca, Nelson et al. 2002). The WCT index (Table 2S) was calculated according to the following formula (Błażejczyk and Kunert 2011):

$$\begin{array}{l}WCT=13.12+0.6215\times\:t-11.37\times\:\left(v10\right)^{0.16}+0.3965\times\:t\times\:\left(v10\right)^{0.16}\end{array}\:$$

where: t – air temperature, v10 – wind speed at 10 m a.g.l.

The second biometeorological index used is insulation predicted (Iclp). It is used to determine the insulation capacity of clothing that atmospheric conditions require in order to maintain the thermal equilibrium of the human body (Błażejczyk 2004). This index includes the formula of Burton and Edholm (1955) and determines the total thermal insulation of clothing and its surrounding thin, near-surface air layer. It also includes the formula of Fourt and Hollies (1970), which determines the thermal insulation of the near-surface air layer (Błażejczyk and Kunert 2011):

$$\begin{array}{l}Iclp=\left(\frac{0.082\times\:(91.4-\left(1.8\times t+32\right)}{0.01724\times\:M}\right)-\left(\frac1{0.61+\left(1.9v\right)^{0.5}}\right)\end{array}$$

where: t – air temperature, M – metabolism, v – wind speed.

Insulation predicted is expressed in clo units. A value of 1 clo corresponds to a thermal resistance of 0.155 K·m^˗2^·W^˗1^ (ISO 9920 2007). A set of clothing that guarantees such thermal resistance – e.g. typical work clothes – provides thermal comfort to a person at an ambient temperature of 21 °C at a wind speed of up to 0.1 m·s^˗1^ and relative humidity of no more than 50%. In this study, the Iclp calculations assumed metabolism values of 70 and 135 W·m^˗2^ for a standing human and a human moving at a speed of 4 km·h^˗1^, respectively. For bioclimatic purposes, it is assumed, following Krawczyk (1993), that thermal resistance values of clothing are: <0.5 clo – very light summer clothing; 0.5–1.0 clo – light summer clothing; 1.0–1.5 – ordinary summer clothing with accessories that increase its thermal insulation; 1.5–2.5 clo – spring/autumn clothing; 2.5–3.0 clo spring/autumn clothing with increased thermal insulation. Winter clothing is characterized by thermal insulation of 3.0–4.0 clo, and > 4.0 clo indicates heavy winter clothing (“arctic clothing”) used in severe winter conditions.

Additionally, the stimulating effect of thermal conditions was determined based on air.

temperature variability. The proposal of Bajbakova et al. 1963) was used, but instead of the day-to-day variability of daily means, we present day-to-day changes in air temperature between individual observations taken at 14:00 LT or 15:00 LT (for the historical and contemporary periods, respectively).

The proposal of Bajbakova et al. (1963) was used, but instead of the day-to-day variability of daily means, the day-to-day changes in temperature at 14:00 LT were presented. The relationship between these changes and the intensity of thermal stimuli is as follows: ≤ 2.0 °C neutral, 2.1–4.0 °C perceptible, 4.1–6.0 °C significant and ≥ 6.1 °C severe.

## Results

### General meteorological conditions

**Fig. 3:**
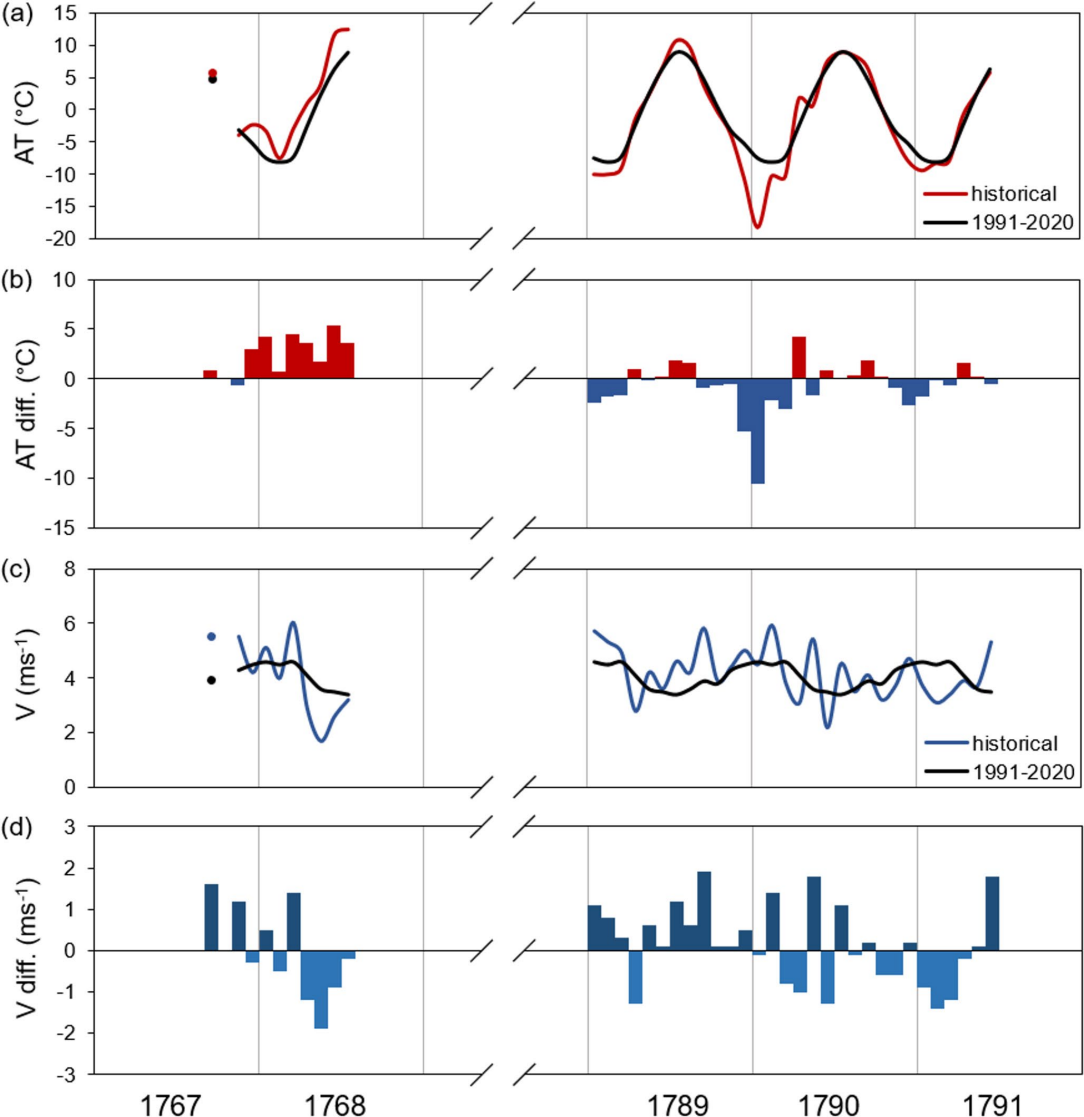
The courses of: **a** monthly mean air temperature (AT); **c** wind speed (V, 1.2 m a.g.l.); and (**b** and **d**) their differences between historical and contemporary periods (AT diff. and V diff., respectively) at Nuuk. Note: historical period = Sep 1767–Jul 1768, Jan 1789–Jun 1792 (at 14.00 LT); contemporary period = 1991–2020 (at 15.00 LT)

At Nuuk during the historical period (1767–68, 1789–90, 1790–91 and 1791–92), the mean monthly air temperatures at 14.00 LT ranged from ˗18.2 °C in January 1791 to 12.5 °C in July 1768 (Table 1). Instantaneous values in the afternoon hours ranged from ˗27.4 °C (10 February 1791) to 17.8 °C (8 and 9 June 1768). In the contemporary period at 15:00 LT, over the annual cycle, mean monthly values (8.9 and 8.2 °C) were highest in July and August, respectively, and lowest (˗8.2, ˗7.6 and ˗7.4 °C) in February, January and March, respectively. Additionally, Table 1 shows a comparison of early afternoon temperature with average temperatures from the contemporary period. In Nuuk, the average annual air temperature in the years 1991–2020 was ˗1.0 °C. Over the annual cycle, average air temperatures were lowest in February (˗8.3 °C) and highest in July (7.0 °C) (Table 1, see also Cappelen and Drost Jensen 2021). The table shows that, in winter in the Arctic regions, the daily mean values and those at 15:00 LT are very similar, which results from the daily mean course of air temperature being very weakly expressed. This fact has been emphasized for the Arctic areas by, among others, Przybylak (1992) and Araźny (2008). The differences between mean diurnal temperatures and those at 15:00 LT are greatest in summer (e.g., July, at approx. 2 °C) (Table 1). In contemporary times, extreme temperatures at Nuuk ranged from ˗26.6 to 24.0 °C (Cappelen and Drost Jensen 2021). At Nuuk, the contemporary period 1991–2020 was warmest of the period 1784–2020, and the decade 2001–2010 was warmer than any other 10-year period (Cappelen 2021). At Nuuk, the largest monthly air temperature difference (˗10.6 °C) between the historical and contemporary periods was observed in January 1790 (Fig. 3). Comparing the period of the year for which data exists for both the historical and contemporary period (i.e., January–June), the average afternoon temperature was about ± 0.2–2.0 °C lower than at present in the years 1790–92, but significantly higher (about 3.4 °C) in the expedition year 1767–68 (Table 1).

**Table 1:** Average monthly and seasonal air temperature and wind speed (1.2 m a.g.l.) at Nuuk in historical (1767–68, 1789–92) and contemporary (1991–2020) periods

Period	Air temperature [°C]						Wind speed [m∙s^˗1^]					
	1767–68	1789–90	1790–91	1791–92	1991–2020	1991–2020*	1767–68	1789–90	1790–91	1791–92	1991–2020	1991–2020*
	14:00 LT				15:00 LT	Mean	14:00 LT				15:00 LT	Mean
Sep	5.7		4.0	6.7	4.9	3.9	5.5		5.8	4.1	3.9	3.7
Oct			˗0.1	0.8	0.6	0.1			3.9	3.2	3.8	3.9
Nov	˗3.9		˗3.7	˗4.1	˗3.2	˗3.3	5.5		4.4	3.7	4.3	4.4
Dec	˗2.3		˗10.6	˗7.9	˗5.3	˗5.3	4.2		5.0	4.7	4.5	4.4
Jan	˗3.3	˗10.0	˗18.2	˗9.4	˗7.6	˗7.7	5.1	5.7	4.5	3.7	4.6	4.6
Feb	˗7.5	˗10.0	˗10.3	˗8.3	˗8.2	˗8.3	4.0	5.3	5.9	3.1	4.5	4.5
Mar	˗2.9	˗9.1	˗10.4	˗8.0	˗7.4	˗7.8	6.0	4.9	3.8	3.4	4.6	4.5
Apr	1.0	˗1.6	1.7	˗1.0	˗2.6	˗3.2	2.9	2.8	3.1	3.9	4.1	4.1
May	4.0	2.2	0.6	2.5	2.3	1.1	1.7	4.2	5.4	3.7	3.6	3.5
Jun	11.7	6.5	7.2	5.8	6.3	4.7	2.6	3.6	2.2	5.3	3.5	3.3
Jul	12.5^#^	10.7	8.9		8.9	7.0	3.2^#^	4.6	4.5		3.4	3.2
Aug		9.8	8.5		8.2	6.7		4.2	3.5		3.6	3.3
Sep–Nov			0.1	1.2	0.7	0.2			4.7	3.7	4.0	4.0
Dec–Feb	˗4.4		˗13.1	˗8.5	˗7.0	˗7.1	4.4		5.1	3.8	4.5	4.5
Mar–May	0.7	˗2.8	˗2.7	˗2.2	˗2.6	˗3.3	3.5	4.0	4.1	3.7	4.1	4.0
Jun–Aug		9.0	8.2		7.8	6.1		4.2	3.4		3.5	3.3
Jan–Jun	0.5	˗3.7	˗4.9	˗3.1	˗2.9	˗3.5	3.7	4.4	4.1	3.8	4.2	4.1

Explanation: ^#^ – data available for 1–22; * averages according to Cappelen and Drost Jensen (2021)

The degree of biometeorological impact of the thermal environment can be assessed in detail on the basis of the magnitude of day-to-day changes in air temperature. The distribution of biometeorological stimuli in the annual cycle, determined on the basis of changes in air temperature, is shown in Fig. 1S. In Nuuk in the contemporary period, on average, neutral stimuli occur with the highest frequency over the annual cycle (53%). These stimuli vary over the course of the year from 47% in June and July to 72% in September. Human well-being was recorded as “perceptible” and “significant” on 27% and 12% of days per year on average, respectively. Severe, high-intensity stimuli are very significant to humans, as they are distressing. These stimuli occurred on 8% of days in Nuuk in the contemporary period. In the summer half-year, severe stimuli rarely occur, but in winter and early spring they can constitute up to 26% of all thermal stimuli. However, in the historical period, the annual cycle was similar to that observed today. However, analysing individual months in individual years, exceptions to this rule are found, as in February 1791. At that time, severe stimuli accounted for as many as 46% of all cases. This was due to very dynamic weather changes in this month. For example, on February 10–12, 1791, a rise in air temperature was observed (from ˗27.4 to ˗18.0 and ˗5.6 °C, respectively).

From a bioclimatic perspective, information on wind speed is also important. During the historical period, in the 18th century, mean monthly wind speeds at 14.00 LT ranged from 1.7 m·s^˗1^ in May 1768 to 6.0 m·s^˗1^ in March 1768. Meanwhile, at the turn of the 21 st century the average wind speed around noon over the annual cycle ranges from 3.4 m·s^˗1^ (in July) to 4.6 m·s^˗1^ (in January and March), with an annual average of 4.0 m·s^˗1^. Comparing the average values from the contemporary period (from 15:00 LT and for the whole day) there are small differences of about ± 0.2 m·s^˗1^ for individual months (Table 1). However, comparing the average monthly wind speed at Nuuk between the historical and contemporary periods, the largest differences of ± 1.9 m∙s^−1^ were observed in September 1789 and May 1768, respectively (Fig. 3). Analysis of the shared period (January–June) showed that, in historical and contemporary times, the mean afternoon wind speed was about ± 0.1–0.5 m·s^˗1^ lower in the years 1767/68 and 1790–92 than in the present and slightly higher (0.2 m·s^˗1^) in 1790 (Table 1).

### Biometeorological conditions

#### Wind chill temperature

At Nuuk, the annual mean WCT value was ˗5.0 °C at 15:00 LT during the period 1991–2020. Over the annual cycle, the mean monthly WCT values ranged from the range ˗11.4÷˗10.5 °C (meaning moderate risk of frostbite) in the period from January to March, to the range 7.9 ÷ 8.8 °C (no risk of frostbite) in July–August (Table 2). Average monthly values above 0 °C, corresponding to no risk of frostbite, were recorded from May to September. However, perception of cold (< 0 °C WCT) occurred on approximately 58% of days of the year. In the contemporary period, the WCT was lowest (˗34.8 °C) on March 3, 1997, which meant that the risk of frostbite was very high. This means that exposed skin can freeze in 10 to 30 min. The observed air temperature was ˗25.9 °C and wind speed was 7.4 m∙s^˗1^.

**Table 2:** Average monthly and seasonal WCT (°C) at Nuuk in historical periods, and average monthly WCT between 1991 and 2020 with monthly maximum and minimum WCT in the afternoon hours

Period	1767–68	1789–90	1790–91	1791–92	1991–2020		
					Mean	Max	Min
	14:00 LT				15:00 LT		
Sep	4.5		2.9	6.4	4.0	8.0	1.9
Oct			˗0.7	0.5	˗0.8	2.7	˗4.5
Nov	˗7.0		˗5.4	˗5.6	˗5.4	0.4	˗9.3
Dec	˗4.5		˗13.8	˗9.9	˗8.0	˗2.2	˗13.5
Jan	˗6.0	˗13.7	˗22.3	˗11.5	˗10.6	˗3.7	˗19.6
Feb	˗9.8	˗13.4	˗13.7	˗9.8	˗11.4	˗1.8	˗22.3
Mar	˗5.9	˗12.1	˗12.9	˗9.8	˗10.5	˗4.1	˗18.3
Apr	0.8	˗2.7	1.0	˗2.2	˗4.6	˗0.4	˗10.4
May	5.1	1.0	˗1.5	1.7	1.1	6.0	˗4.2
Jun	12.5	6.2	7.8	5.2	5.8	8.8	0.9
Jul	13.2*	10.7	8.8		8.8	10.9	6.2
Aug		9.7	8.6		7.9	10.9	5.7
Sep–Nov			˗1.1	0.5	˗0.7	3.7	˗4.0
Dec–Feb	˗6.7		˗16.6	˗10.4	˗10.0	˗2.6	˗18.5
Mar–May	0.0	˗4.6	˗4.5	˗3.5	˗4.7	0.5	˗11.0
Jun–Aug		8.9	8.4		7.5	10.2	4.2
Jan–Jun	˗0.5	˗5.8	˗6.9	˗4.4	˗5.0	0.8	˗12.3

Explanation: * – data available for 1–22

**Fig. 4:**
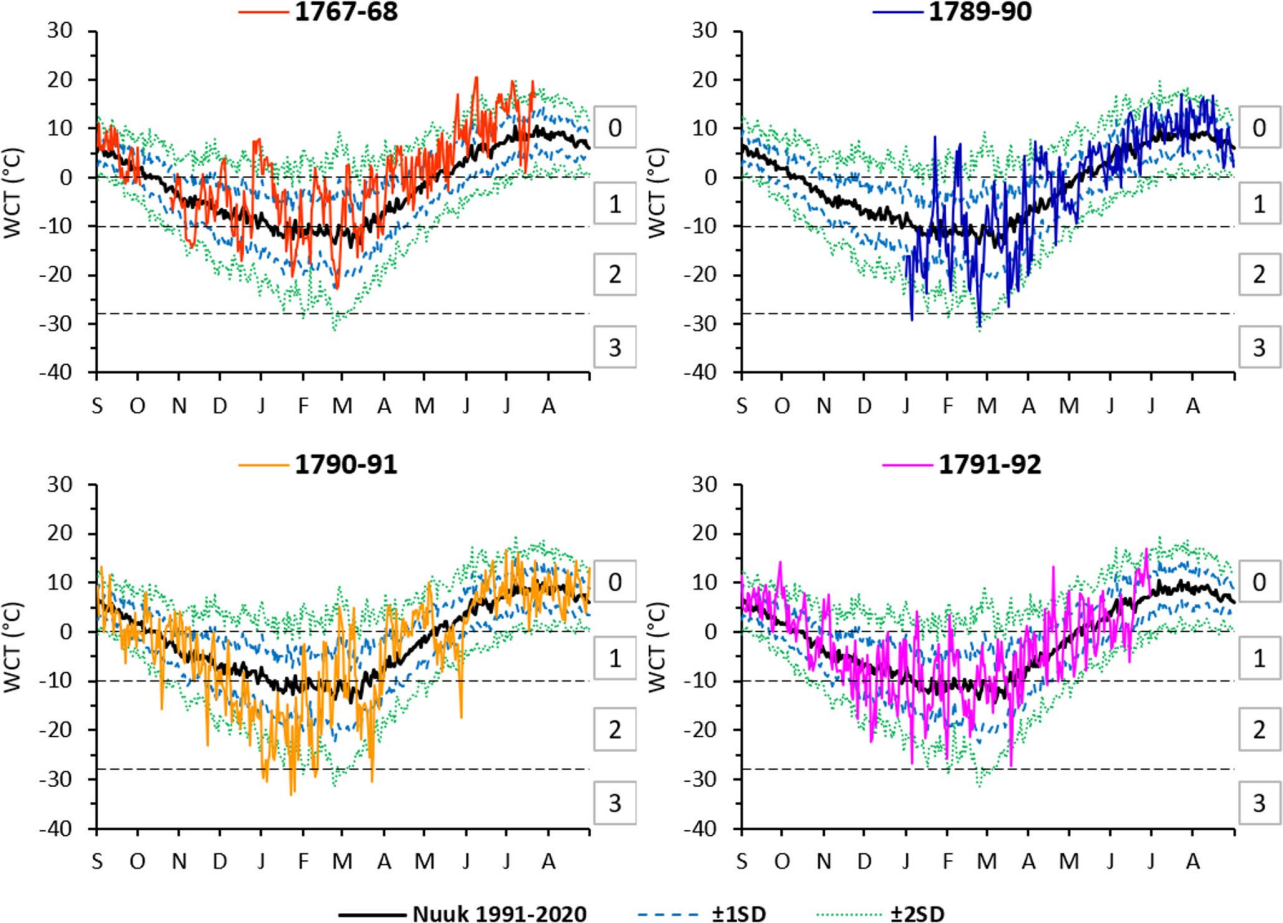
Annual courses of average diurnal WCT in the historical and contemporary periods (from the Nuuk station). Standard deviations (± 1 SD and ± 2 SD) were calculated for the contemporary period 1991*–*2020. Explanation: risk of frostbite: 0 – lack, 1 –low, 2 – moderate, 3 – high

In Nuuk, WCT values were lower during the years 1790–91 and higher in 1768 and 1792 as compared to the contemporary period (for the period January–June). In particular, 1768 was 4.5 °C milder for humans than the conditions prevailing today (Table 2). During the 18th century, the worst conditions for humans according to WCT (˗22.3 °C, meaning moderate risk frostbite) were recorded in January 1791. At that time, there was a negative WCT anomaly (by 11.7 °C) relative to the contemporary period. WCT values as low as the aforementioned are also observed in Nuuk in winter today. In the historical period, the highest monthly mean WCT (13.2 °C, i.e. without the risk of frostbite) occurred in July 1768 (Table 2). In the analysed historical period, the lowest WCT value (˗33.1 °C) occurred on January 23, 1791, which meant that the risk of frostbite was very high. The air temperature was then measured at ˗25.2 °C and the wind speed was 6.1 m∙s^˗1^. The WCT values at 14.00 LT on individual days at the end of the 18th century generally do not deviate by more than ± 2 SD from the average for the years 1991–2020 (Fig. 4).

#### Insulation predicted

**Fig. 5:**
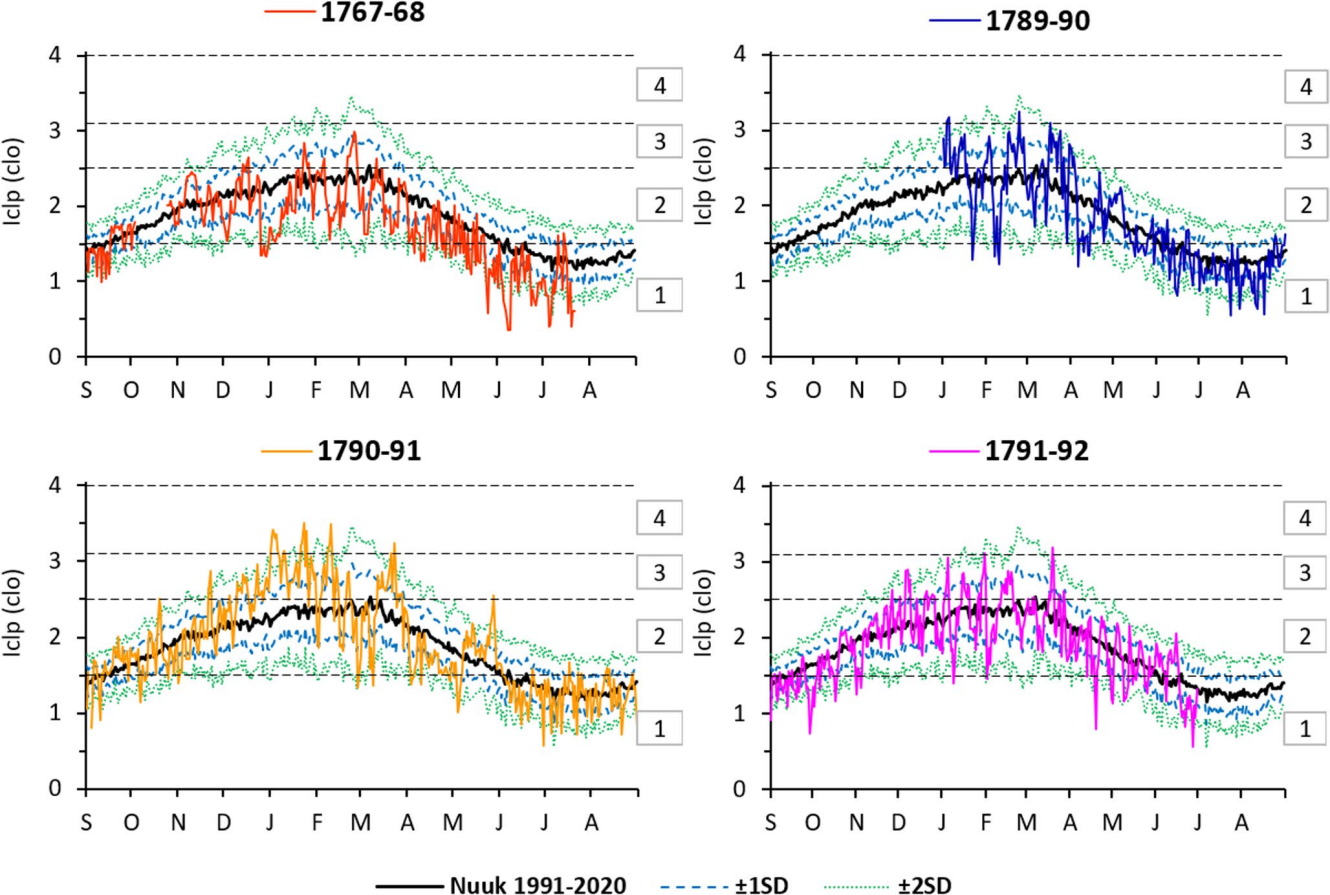
Annual courses of average diurnal Iclp (for a metabolism of 135 W·m^˗2^) in the historical and contemporary periods (from the Nuuk station). Standard deviations (± 1 SD and ± 2 SD) were calculated for the contemporary period 1991*–*2020. Explanation: type of clothing: 1 – summer clothing, 2 – spring and autumn clothing, 3 – spring and autumn clothing with increased thermal insulation, 4 – normal winter clothing

To protect against excessive cooling of the body, it is necessary to use clothing with appropriate thermal insulation. In Nuuk in the contemporary period, i.e. in the years 1991–2020, clothing with significant thermal insulation properties was necessary for humans to achieve thermal comfort during movement (metabolism = 135 Wm^˗2^). At the turn of the 21 st century, the annual mean monthly Iclp values ranged from 1.3 clo in July and August to 2.4 clo in February (Table 3). Analyzing all days in the contemporary period, the absolute range of Iclp was from 0.43 clo (June 9, 2016) to 3.56 clo (March 3, 1997). In Nuuk, from June to August, it was recommended to wear summer clothing (up to 1.5 clo) in the afternoon. However, theoretically, clothing with thermal insulation of 1.5–3.0 clo, i.e. transitional seasons with increased thermal insulation, should be sufficient for the rest of the year. On some days, in order to ensure thermal comfort of the body, winter clothing is required (> 3.0 clo) (Fig. 5).

**Table 3:** Average monthly and seasonal Iclp (clo) at Nuuk in historical periods, and average monthly Iclp between 1991 and 2020 with monthly maximum and minimum Iclp for a person with metabolism 135 Wm^˗2^

Period	1767–68	1789–90	1790–91	1791–92	1991–2020		
					Mean	Max	Min
	14:00 LT				15:00 LT		
Sep	1.5		1.5	1.4	1.5	1.6	1.3
Oct			1.8	1.7	1.8	2.0	1.6
Nov	2.1		2.0	2.0	2.0	2.3	1.7
Dec	2.0		2.5	2.3	2.2	2.5	1.9
Jan	2.1	2.5	3.0	2.4	2.3	2.8	1.9
Feb	2.3	2.5	2.5	2.3	2.4	3.0	1.8
Mar	2.0	2.4	2.5	2.3	2.3	2.7	2.0
Apr	1.7	1.9	1.7	1.9	2.0	2.3	1.8
May	1.4	1.7	1.8	1.6	1.7	2.0	1.4
Jun	1.0	1.4	1.3	1.4	1.4	1.7	1.3
Jul	0.9*	1.1	1.2		1.3	1.4	1.1
Aug		1.2	1.2		1.3	1.4	1.1
Sep–Nov			1.8	1.7	1.8	2.0	1.5
Dec–Feb	2.1		2.7	2.3	2.3	2.2	1.7
Mar–May	1.7	2.0	2.0	1.9	2.0	2.5	1.8
Jun–Aug		1.2	1.2		1.3	2.8	1.9
Jan–Jun	1.7	2.0	2.1	2.0	2.0	2.4	1.7

Explanation: * – data available for 1–22

Analyzing the historical period (the common period January–June) in the second half of the 18th century in 1790–92, a local inhabitant required very similar thermal clothing insulation as today (Table 3). Only in 1768 did the weather require that a person on the move wear about 0.3 clo less clothing than in contemporary times. During the years 1767–92, the monthly mean values of Iclp ranged from 0.9 clo in July 1768 to 3.0 clo in January 1791 (Table 3). Looking at all days in the historical period, the demand for Iclp ranged from 0.36 clo (8 June 1768) to 3.51 clo (23 January 1791).

Table 3; Fig. 5 show the Iclp values for a person moving at 4 km·h^˗1^. However, for a standing person to maintain thermal comfort, clothing with twice as much thermal insulation is required (Fig. 2S). This is because, during this physical activity, the metabolic heat produced drops to 70 W·m^˗^². This represents a decrease of heat about 100% compared to the value produced by a person moving at a speed of 4 km·h^˗1^ (Araźny 2006). During the years 1991–2020, annual Iclp values ranged from 2.6 clo in July and August to 4.8 clo in February. These values indicate that thermal comfort in a standing position in Nuuk required winter clothing with significant thermal insulation properties for 9 months of the year. The highest momentary demand for clothing ensuring thermal comfort while standing in the study area theoretically reached 7.03 clo (3 March 1997). However, in the historical period (January–June) in the years 1790–92, the demand for thermal insulation of clothing for a person standing was a maximum of 6.94 clo, as on 23 January 1791. Values of > 4 clo mean the demand for so-called “heavy arctic” winter clothing. This consists of, among other things, fur or a coat with fur lining. Gloves, headgear and footwear of fur, plus other items of clothing as in the transitional seasons (Krawczyk 1993).

## Discussion

In the 20th and 21st centuries, dozens of different indexes were created to assess the impact of the atmospheric environment on humans. Most have no direct reference to the physiological reactions occurring in the body under the influence of changing thermal conditions in the environment. In the 1990 s, “multi-node” models of human heat balance were developed that describe all the complex mechanisms of the body’s heat management. Based on one of these models, a new thermal index (UTCI – Universal Thermal Climate Index) was created to assess human thermal loads (Jendritzky et al. 2012; Błażejczyk 2013). To determine the UTCI value, human heat balance is calculated for given meteorological conditions, taking into account (in addition to air temperature and humidity) wind speed and mean radiant temperature (Tmrt). For weather conditions during historical periods of the 18th century, the only information we have relates to air temperature and descriptions of wind force. Therefore, this work can only use biometeorological indexes that are based on those two parameters. Hence, it was decided to use WCT and Iclp.

Wind chill temperature is a function of both wind speed and air temperature and was developed to straightforwardly represent the decrease in air temperature the humans experience on exposed skin due to the flow of cold air (Oczevski and Bluestein 2005). In the Arctic, WCT in historical times at the turn of the 20th century in Franz Josef Land has been analyzed by Araźny et al. (2019). WCT indicates that the conditions that prevailed in Franz Josef Land were significantly less comfortable (risk of frostbite all year round) than in the area analysed in this study. The issue of WCT in the second half of the 18th century in Labrador has bewen addressed by Chmist et al. (2025). They found that bioclimatic conditions were more favourable for humans in historical times than in the years 1991–2000. According to Chmist et al. (2025), frostbite risk to exposed parts of the body in the historical period at the Nain station was absent only in August, whereas in Okak it was absent in both July and August. However, in the comparative period (1991–2000) the risk of frostbite was absent in July and August. WCT was also analysed in the Norwegian Arctic (Araźny 2008) at six meteorological stations in the years 1971–2000. The author found that, in the Norwegian Arctic, it felt cold (< 0 °C WCT) on 73% of the days of the year. The fewest such days were recorded in Jan Mayen (68%) and the most in Hopen (81% of days). Researchers who have conducted WCT research in North America include: Keimig and Bradley (2002), Mekis et al. (2015), Howarth and Laird (2017) and Kim et al. (2025). The results of these studies show that WCT values were steadily increasing in the late 20th and early 21 st centuries, which indicates an improvement in human thermal sensations. This allows for, among other things, increased ability to perform outdoor activities. These changes are primarily attributed to higher air temperatures rather than changes in wind speed (Howarth and Laird 2017; Kim et al. 2025).

In variable and cold climatic conditions, balance between the amount of heat gained and lost by the human body is maintained through adaptive thermoregulatory reactions, but also thanks to properly selected clothing (Krawczyk 1993; Yan and Oliver 1996). Clothing used in the Arctic has changed over the centuries (Svensson 1992). Nowadays, clothing can be optimized for thermal comfort in each type of environment and type of physical activity. Insulation predicted is an index that can be used to determine and compare the thermal insulation of clothing needed in given meteorological conditions to maintain the body’s thermal equilibrium. For the historical period in the Arctic, Iclp data are available for the First International Polar Year (1882–83, hereinafter IPY) (Araźny 2010). In this work, Iclp was calculated for nine Arctic stations, and this historical period was compared against the period 1971–2000 for the Jan Mayen station (Norwegian Arctic). During the first IPY, the thermal insulation properties of clothing for a person on the move had to be about 0.1 clo higher than at the end of the 20th century. Regarding the requirements for clothing with optimal insulation for persons engaged in activity in the first IPY, especially in the winter months, heavy Arctic clothing was necessary (Araźny 2010). Another work from the historical period from the Arctic is the study of Franz Josef Land by Araźny et al. (2019). Meteorological conditions in this area indicate that the thermal insulation requirements for clothing in 1899/1900 and 1913/14 were similar to the contemporary period (1981–2010). In 1903/04, there was a 0.3 clo increase in clothing insulation required to achieve thermal comfort, whereas in 1930/31 there was a decrease (by 0.3 clo) compared to the period 1981–2010 (Araźny et al. 2019). For the historical period, there is another study by Chmist et al. (2025). In the 18th century in Labrador, according to the Iclp, sets of clothing at least 2.5 clo were required mainly from December to March, while summer clothes were allowed from May to September. The authors state that, in the contemporary period (1991–2000), the period in which summer clothes were used covered a shorter period of June–September. In the Norwegian Arctic, Iclp has been analysed for the period 1971–2000 by Araźny (2006, 2008). He noted that, during this period in the Norwegian Arctic, the requirement for thermal insulation of clothing gradually decreased, which is a result of climate change, i.e. warming.

## Conclusions and Final Remarks

The main results for Nuuk in the present paper can be summarized as follows:

- The afternoon air temperature in historical times (in the years 1790–92) was 0.2–2.0 °C lower than at present. However, in the expedition year 1767–68, the temperature was significantly higher (by about 3.4 °C) than in the contemporary period (Table 1).
- The mean afternoon wind speed was about ± 0.1–0.5 m·s^˗1^ slower in the years 1767–68 and 1790–92 than at present and slightly higher (0.2 m·s^˗1^) in the year 1789–90 (Table 1).
- The degree of biometeorological impact of the environment (according to day-to-day air temperature changes) indicates a predominance of neutral stimuli (> 50%) in the contemporary period. On the other hand, severe stimuli of high intensity that have a distressing effect on humans were noted in 8% of cases. The average four-year historical period was characterized by a similar breakdown of biometeorological stimulus categories to the present one (Fig. 1S).
- In the contemporary annual cycle, average monthly WCT values indicate a range of frostbite risk from moderate in the period from January to March to zero from May to September. Compared to the contemporary period, WCT values were lower during the years 1790–91 and higher in the years 1767/68 and 1791/92 (Table 2).
- Average monthly Iclp values during 1991–2020 ranged from 1.3 clo in July and August to 2.4 clo in February for a person in motion. In the years 1790–92, thermal insulation of clothing similar to that used today was required to maintain thermal comfort. The exception was 1768, when the requirement was about 0.3 clo lower than today (Table 3).

We acknowledge that our study has certain limitations that impact the degree of certainty with which conclusions can be drawn. Firstly, in the absence of direct measurements of wind speed in m∙s^−1^, a degree of uncertainty results from the use of statistical calculations. Secondly, past meteorological data are often incomplete, irregular or derived from instruments of varying quality. Thirdly, our study is limited to a historical period of relatively few years of research.

In terms of applicability, the study is nonetheless extremely valuable because it is the oldest data available for this region of the Arctic and thus contributes to understanding the extent of natural changes in bioclimatic conditions over a longer time period. In the Arctic, human impacts on the climate and environment were minimal before 1950 (Przybylak 2000), and the early instrumental period is considered to have been before 1890 (Brönnimann et al. 2019). In the Arctic, the 21 st century regional warming, from a human perspective, is unprecedented among recorded observations. Understanding the variability of bioclimatic conditions in the Arctic is particularly important for several reasons: for human safety (including the risk of frostbite and hypothermia), for planning life in polar conditions and fieldwork (including appropriate clothing, work pace, and breaks), and for assessing risk in extreme weather situations (through understanding the range of extreme weather and bioclimatic conditions). 

## Supplementary Information

Below is the link to the electronic supplementary material.

ESM 1 DOCX (232 KB)

## Data Availability

The data presented in this research are available on request from the corresponding author and data from the Danish Meteorological Institute are available from https://www.dmi.dk.
