# Supplementary material for: Comparative study of bioclimatic conditions in southwestern Greenland between the late 18th century and the present
Source: Int J Biometeorol. 2026 Mar 4;70(3):82. doi: 10.1007/s00484-026-03132-5 (PMC12960345; doi:10.1007/s00484-026-03132-5)
Supplement: Supplementary file 1 — DOCX (232 KB) [file 484_2026_3132_MOESM1_ESM.docx]

**Table 1S** Example of descriptions of the strength of the wind used by the Moravian missionaries in 1767-68 (after Demarée and Ogilvie 2008)

| Scale | Description |
| --- | --- |
| 1 | a very light wind that only ripples the water |
| 2 | a topsail breeze, or a light wind in which one may hoist all sails on a ship |
| 3 | a marchsail breeze, or a fresh wind where half the sails on a ship must be taken down |
| 4 | a strong wind |
| 5 | a weak storm |
| 6 | a heavy storm |

## **Table 2S** Characterization of bioclimatic conditions based on WCT

| WCT  [°C] | Risk of frostbites | Health concern |
| --- | --- | --- |
| 0  to -9.9 | Low risk | Slight increase in discomfort |
| ˗10.0  to ˗27.9 | Moderate risk | Uncomfortable. Risk of hypothermia if outside for   long periods without adequate protection. |
| ˗28.0  to ˗39.9 | High risk | Exposed skin can freeze in 10 to 30 minutes. Check face and extremities for numbness or whiteness. High risk of hypothermia if outside for long periods without adequate clothing or shelter from wind and cold. |
| ˗40.0  to ˗47.9 | Very high risk | Exposed skin can freeze in 5 to 10 minutes*. Check face and extremities for numbness or whiteness. Very high risk of hypothermia if outside for long periods without adequate clothing or shelter from wind and cold. |
| ˗48.0  to ˗54.9 | Severe risk | Exposed skin can freeze in 2 to 5 minutes*. Check face and extremities frequently for numbness or whiteness. Severe risk of hypothermia if outside for long periods without adequate clothing or shelter from wind and cold. |
| ˗55  and colder | Extreme risk | Exposed skin can freeze in less than 2 minutes.* Danger!  Outdoor conditions are hazardous. |

*In sustained winds over 50 kmh^˗1^, frostbite can occur faster than indicated, (<https://www.canada.ca/en/environment-climate-change/services/weather-health/wind-chill-cold-weather/wind-chill-index.html#toc0>)


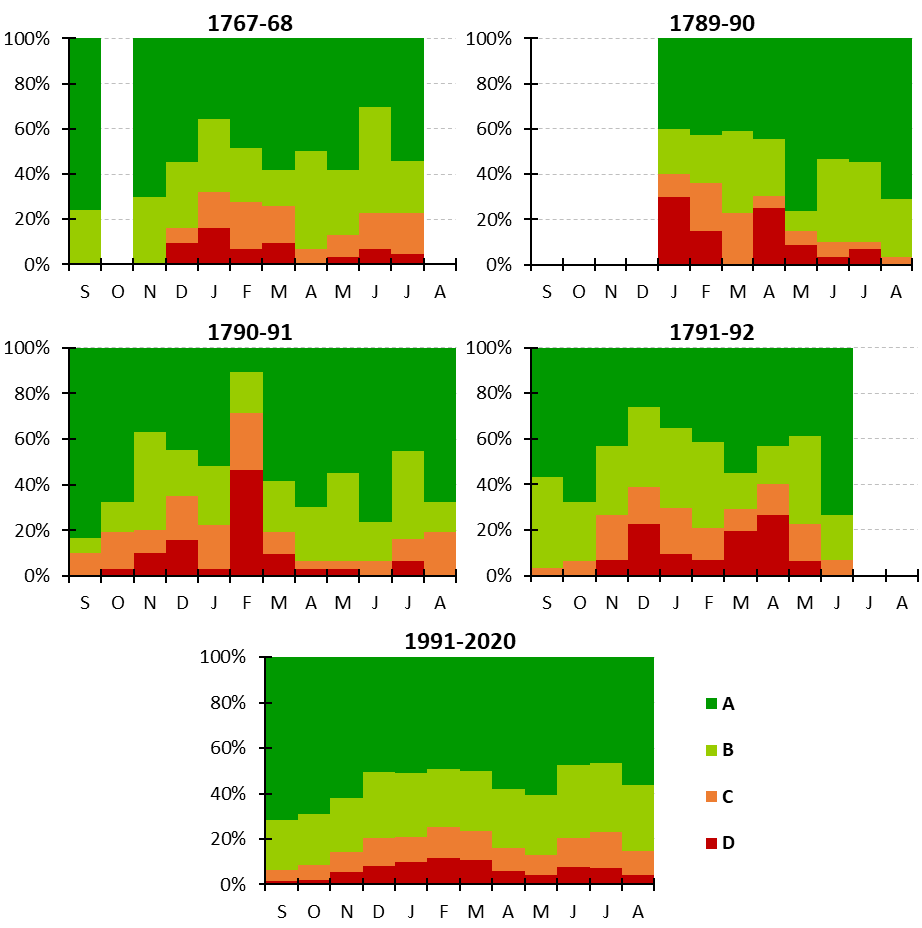


**Fig. 1S** Relative frequency of temperature stimuli based on day-to-day changes in air temperature in the annual course in particular historical periods and for average from present period (1991*–*2020) at Nuuk. Explanation: strength of stimuli: A – neutral, B – perceptible, C - significant, D - severe


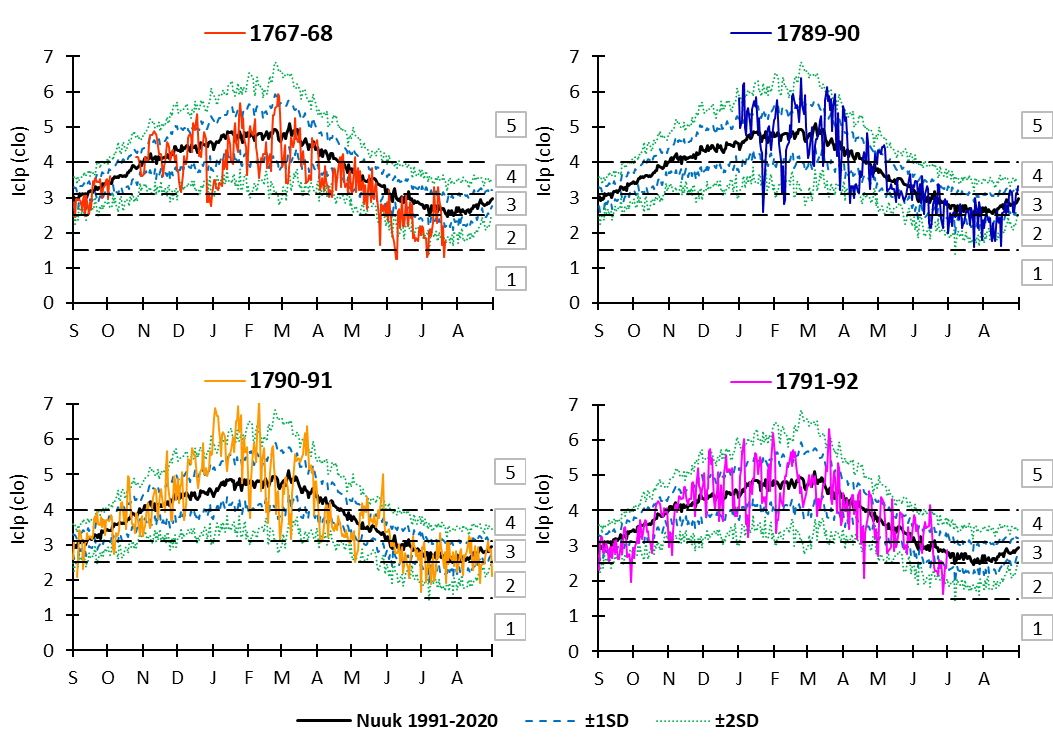


**Fig. 2S** Annual courses of average diurnal Iclp (for a metabolism of 70 W/m^2^) in the historical and contemporary periods (from the Nuuk station). Standard deviations (±1 SD and ±2 SD) were calculated for the contemporary period 1991*–*2020. Explanation: type of clothing: 1 – summer clothing, 2 – spring and autumn clothing, 3 – spring and autumn clothing with increased thermal insulation, 4 – normal winter clothing, 5 – arctic clothing
